# On Diseases of the Urinary Organs

**Published:** 1839

**Authors:** 


### ON DISEASES OF THE URINARY ORGANS.

I. Urinary Diseases and their Treatment. By *Robert Willis*, M.D., &c. &c.

III. Traité des Maladies des Reins, &c. Par *P. Rayer*.

[Continued.]

In our last article,* we presented the views of Dr. Christison on the important subject of albuminous urine. Those views occupied (for they were elaborate and precise) the whole space which we could devote to urinary disorders, and necessarily diverted us from the pages both of M. Rayer and of Dr. Willis. We return to those gentlemen, and shall pursue, we will not say complete, the examination of the maladies of which they treat.

We have observed upon a previous occasion that it is difficult to amalgamate the observations of M. Rayer with those of the other authors who have been before us. The best plan appears to us to take that author who is most copious or most correct on any particular point, and, making his page the text for the time, to collate with it what remarks on the part of another may seem calculated to correct or amplify it.

Pursuing the arrangement and order adopted by Dr. Willis, we may still occupy ourselves with those :—

### Morbid States in which Certain Matters, being Constituents of the Blood, are contained in the Urine.

1. Of those morbid states, sero-albuminous, or, as it is commonly called, albuminous urine is the most important. That has been already amply discussed, and we turn to that condition of the urine, in which it contains the oily and albumino-fibrinous elements of the blood.

### 2. *On Oleo-Albuminous Urine.*†

*a.* Instead, says Dr. Willis, of a simple albuminous impregnation, there are numerous cases on record in which the urine has been secreted with such an addition of oleaginous particles that it has borne the closest resemblance to milk, or to water with which a quantity of cream had been mingled. Such a state has been observed to occur in the course of certain diseases.

*Case.* A gentleman, after a succession of long journeys, in the course of which he enjoyed excellent health, returned home in the same satisfactory state. A few days after his arrival, however, he was attacked with

* Vide No. 60. Pp. 349—397.

† "Chylous urine" of Prout.

slight diarrhœa, and a trifling catarrhal affection of the chest; and by-and-by he perceived that his urine, which was rather more scanty than usual, was of a milky-white colour, but passed without any uneasiness. On standing, this urine, which had no peculiar smell and reddened litmus paper, let fall a precipitate of a white cheesy-looking substance, amounting nearly to one half of the whole quantity of fluid evacuated. Intercepted on a filter and washed with distilled water the deposite was found to have all the properties of caseum, or coagulated albumen, becoming transparent and horny when dried, being insoluble in alcohol, swelling up and turning gelatinous when digested in acetic acid, and forming a deep violet-blue coloured solution when treated with hydrochloric acid. Heat applied to the urinous fluid, freed from the deposite by filtering, threw down an additional precipitate of albumen, which yielded a considerable quantity of oily matter when treated by boiling alcohol. Tested farther, the urine was found to contain its usual salts and constituents, and in addition a notable quantity of acetic acid.

This same condition has been observed during the exhibition of certain medicines. Thus M. Chevallier found the urine of a patient under the influence of mercury, which had become white and opaque or milky, to contain a large quantity of albumen mixed with oleaginous matter.

In other instances, no disease would seem to have existed, nor was any powerful medicine in the course of exhibition.

*Case.* A creole of the Isle of France, in the apparent enjoyment of perfect health, was in the habit of passing urine of the whiteness, consistence, and opacity of milk. This fluid was neutral; it had a sweetish saline taste that was not unpleasant. It was coagulable by heat, and the solid substance presented the most perfect analogy to caseous matter. Had this urine been tested more particularly, it would in all probability have been found to owe its whiteness and opacity in great part to the presence of an oleaginous matter.

In these, and in many other similar cases, the opacity of the urine will be seen to have been attributed to the presence of the albuminous principle in the shape of caseine. But M. Rayer doubts the justice of this notion, and disputes the accuracy of the observations which have given birth to it. In a great number of cases which he had had an opportunity of examining in an extensive private practice, and in the ample wards of the Hôpital de la Charité, he had never discovered the true milk-globule in urine, save in a single instance, and in this the patient was detected scheming—she had mixed a quantity of milk with her urine after it was discharged from the bladder. The milky appearance of the urine whenever it occurs, is due to the suspension of oily matter in the fluid impregnated with albumen.

M. Rayer offers the following as a summary of what he thinks established on the subject of "milky urine."

1. The occurrence of milky urine as a natural circumstance,* though generally admitted, is doubted, if not denied, by M. Rayer.

2. The existence of a certain quantity of fatty matter in the urine gives to it a milky appearance.

* That is (we suppose) independently of deception.—*Eds.*

3. Caseine and milk globules have hitherto been only met in urine rendered milky artificially.

4. Urine, really milky, may be easily distinguished from chylous, or oleo-albuminous, or purulent urine, by re-agents and the microscope.

5. Milky urine should contain caseine coagulable by acetic acid, and should present milk globules appreciable by the microscope.

We return to Dr. Willis, and we pass to some modifications of the oleo-albuminous urine.

"But," he observes, "the milky urine hitherto described is not the only variety of this fluid that is encountered. The oleo-albuminous matter is not always suspended as it were in the menstruum, and apt to subside to the bottom when the mixed fluid is allowed to stand for some time at rest. Occasionally it is intimately incorporated with the fluid evacuated, and instead of falling to the bottom as a precipitate, possesses the property of coagulating spontaneously and entangling the whole of the water in its meshes, so as to form one homogeneous tremulous mass like jelly, or the fresh coagulum of the blood. The albuminous principle here obviously presents the distinguishing feature of the fibrinous modification of albumen—spontaneous coagulation. Urine of this description presents itself with every degree of opacity. Sometimes it is of a pearly-white colour and nearly as opaque as fresh-drawn milk; at other times it is almost transparent, of an amber-yellow tint, and in its general appearance very much like orange jelly; in other instances still the colour is reddish, and the mass closely resembles pale red-currant jelly. This singular coagulum by-and-by begins to separate into two portions, exactly as blood does, the one watery or serous, the other, freed from moisture by gentle pressure, firm, elastic, fibrous, and possessed of all the sensible as well as chemical properties of fibrine.

The kind of albuminous urine now described is by no means common, especially in Europe. Different practitioners have, however, met with a case or two of it. I remember Mr. Abernethy used to mention the case in his lectures of a female, who at times discharged urine of such a kind that had it been allowed to cool in a proper shaped vessel might, in so far as appearance went, have been served at table as blanc-mange. This patient Mr. Abernethy met with, after the lapse of a dozen years or so, looking fat and well, enjoying tolerable health, and still occasionally voiding urine of the old description. Dr. Prout, in his 'Inquiry,' has particularly described two cases which occurred in the practice of Dr. Elliotson, in which the urine at one time bore a strong resemblance to blanc-mange, and at another to orange jelly. The same excellent authority, in his Gulstonian Lectures of the year 1831, informs us that, in the course of his practice, or through the kindness of friends, he had seen as many as eight cases of this spontaneously coagulating oleo-albuminous urine. I have myself met with one, in which the coagulum, from the presence of the colouring matter of the blood, resembled pale red-currant jelly, and which from this circumstance I have referred to the section on Hæmaturia, although I believe the renal affection to have been of the same essential description as that in which the urine is milky, and the coagulum white, or yellowish and opalescent." 164.

Dr. Willis relates the particulars of a case, that of M. Da Costa, which he extracts from La Presse Medicale. It is not necessary to go into it.

c. Occasionally oily matter has been found in large quantity in the urine. A man, writes Dr. Willis, was brought to the Hôpital de la Charité in a state of asphyxia from the fumes of charcoal, where he died in the course of the day. The blood in the heart and great vessels, and the urine in the bladder, were alike remarkable from containing a quantity of yellowish oily globules swimming on their surface. Several other cases of a similar kind have been recorded. In a paper on the discharge of fatty matters from the alimentary and urinary passages, Dr. Elliotson has given the case of a lady aged 79, with whose urine about one third of an ounce of oil was discharged daily for some considerable time during her last illness. This is trifling, however, to the quantity of oil which Bachetoni drew off by the catheter from the bladder of a certain noble damsel (virgo quædam nobilis). Here two ounces were discharged at once on two different occasions. But as the lady had taken four ounces of almond oil some days before as an aperient, and was labouring under a variety of hysterical and anomalous symptoms, it is just possible that, wishing to be thought *extraordinarily* ill, she may have imposed upon her physician. The rank of the individual in these cases goes for nothing.

*d.* The occurrence of oleo-albuminous urine would certainly seem to be far from inconsistent with good health. Mr. Abernethy met the subject of his case, after the lapse of a dozen years, looking fat and well. In one of the cases which Dr. Prout has detailed, the urine was positively known to have been secreted with the morbid characters it presented for a period of five years at least. Here the general health was not at all affected; neither were the reproductive functions interfered with, the subject of the observation, a female, having conceived and borne a living child in the interim.

The disease would appear to prevail endemically in certain regions, for example, in Brazil.

*e.* On the treatment, Dr. Willis, though not silent, says nothing.

3. *On the Discharge of Urine having all the Elements of the Blood mingled with it,*—HÆMATURIA.

The discharge of blood or of bloody fluid from the urinary passages is almost always observed either as a sign and sequence of some disease existing in their course, or still more frequently, perhaps, of the presence in some point of their interior, of a calculous concretion. Yet idiopathic hæmaturia undoubtedly occurs, and *that* may be naturally linked with the oleo-albuminous alteration—the two often alternate. Many of those who pass spontaneously-coagulating urine, observe it to be of a muddy amber-yellow, and of a dilute or deeper red-currant jelly-colour at different times, obviously according to the predominance of the several principles—the oil, albumen, fibrine, and red globules, whose presence gives to the discharged fluid its distinguishing characters, especially its colour.

It exists, as an endemical disease, in some countries. This is the case in the Isle of France. M. Salesse, a native practitioner there, states that three-fourths of the children are affected with hæmaturia at one time or another. In these cases the bloody urine is generally observed to alternate with that which is chylous or oleo-albuminous.

"All these endemic hæmaturias I am inclined to refer to the same state of kidney as that which produces the albuminous, oleo-albuminous, or chylous and spontaneously coagulating urine of homogeneous appearance, and various shades of colour, from the pearly white to the currant-jelly red. The colouring matter of the blood in the whole of these cases seems to be entirely accidental; sometimes it is altogether absent; at no time does it exist nearly in the propoition in which the albuminous principle in one or other of its modifications occurs. The albumino-urinous fluid poured out under these circumstances has all the characters of a secretion. The term hæmaturia would probably be advantageously restricted to those cases in which there was simple exudation of blood from some point of the mucous surfaces lining the urinary passages, or direct extravasation from the vessels of the kidney. Here the fluid discharged is less *albumino-urinous*, than a *mixture of blood and urine;* the vital fluid and the excretion only chance to pass off together. In this case the colouring principle of the blood is in large proportion, and along with the fibrine is precipitated from the urine in the shape of a dark brown sediment. Under such circumstances hæmaturia commonly indicates the existence of some special disease of the kidneys or bladder, or is caused by a foreign body, as a calculus, lodging in their interior. It also frequently accompanies the course of a variety of acute and dangerous diseases. The urine in small-pox for instance, is often bloody. So is it in purpura, in typhoid fever of bad type, in scorbutus, in acute anasarca from exposure to cold, &c. Sometimes the blood presents itself in the urine in the shape of small distinct coagula, or with its serum and colouring matter diffused through that fluid, whilst its fibrine is in threads and masses." 174.

The source whence the hæmorrhage occurs must, in the great majority of instances, be the mucous lining of the renal cavities, ureters, or bladder. The latter is the organ that most frequently furnishes the blood. Dr. Willis observes that, in some subjects, it seems to act very much in the same manner as the membrane of the rectum in hemorrhoids, and to relieve itself from time to time by pouring out a quantity of blood, more or less, from its surface.

But, he goes on to state, by far the most common cause of renal, ureteric, and vesical hemorrhage, is either the presence of a foreign body in the pelvis of the kidney, in the course of the ureter, or in the urinary bladder. The kidneys, like so many other glandular parts endowed with offices proper and peculiar to themselves, are but sparingly supplied with nerves of common sensation. Whence it often happens, that extensive structural changes are found to have taken place in their substance, even before mischief to them was apprehended. In the same way they have frequently contained large calculi, the existence of which would hardly have been suspected, but for the discharge of bloody urine after somewhat violent or continued exercise, such as a long walk, a smart ride on horseback, or a drive in a carriage over a rough road. When hæmaturia follows severe pain in the lumbar region, which shoots down into the thigh, causes retraction of the testis, and is accompanied by sickness and vomiting, we know that a calculus has made its escape from the pelvis of the kidney, and is making its way down the ureter, a canal which, as it performs less of a special function, is endowed with a larger share of common sensibility than the kidney. Of the cause and source of the bloody urine, and sometimes of the pure blood, that is passed when there is a calculus in the bladder, there can be no doubt.

Occasionally hæmaturia is an indication of malignant disease of the kidney; occasionally of a fungoid tumor produced from the inner surface of the bladder, and perhaps still more frequently of an enlarged and altered condition of the prostate gland.

Hæmaturia, then, though occasionally an idiopathic or proper disease, is, in the great majority of instances, a symptom. Its presence should always breed a suspicion of mischief in some part of the urinary apparatus. The determination of *its* presence or absence must, of course, be the object and the work of particular investigation.

We pass to the *Treatment*, on which Dr. Willis says comparatively little. Hæmaturia depending on various causes, its nature must be determined in each case, and the remedies chosen accordingly. If plethora obtains, the antiphlogistic regimen, a mercurial followed by a saline purge now and then, cold enemata, light covering of the pelvis, &c. may all be required. It is seldom necessary or advisable to abstract blood generally in hæmaturia; the subjects of the affection are rarely of a constitution to stand this measure. The local abstraction of blood by cupping from the loins or perinæum, by the application of leeches in the latter situation or to the verge of the anus, however, may sometimes be had recourse to with advantage. When the hæmaturia is vicarious of some other flux, the menstrual, for example, or the hæmorrhoidal, the suppressed or impaired one should be, *if possible*, restored, a thing more easy to be recommended than done. Dr. Willis adds :—

" Some of the articles, however, that seem to exert their influence upon the lining membranes of the urinary passages more especially, may sometimes be tried with excellent effect. The uva-ursi in extract, the diosma crenata or buchu, the oil of turpentine, and even the cantharides used cautiously, and in such a way as to secure their tonic, without inducing their stimulating effects, will often produce the best effects. Where there is pain and irritation, opiates, the warm bath, &c. must be had recourse to. Where the disease is plainly connected with a lax and debilitated habit, general tonic medicines, the preparations of iron, bitters, the bark, &c. will be the appropriate remedies.

It sometimes happens, when the discharge of blood is considerable, that the fluid, accumulating in the bladder, coagulates there, and forms a solid mass which it is often extremely difficult to get away. Under these circumstances, we have been recommended to introduce a sound or catheter for the purpose of breaking down the clot. This advice must be acted upon with great caution; much mischief might ensue upon its being enforced by a rash or even a bold hand. It will probably be better to pass as full-sized a catheter as can be made to enter, and by means of a syringe fitting into the outer orifice of the instrument, to endeavour to suck out the coagulum ; or warm water may be repeatedly injected so as to wash down the clot." 181.

The Sixth Chapter of Dr. Willis' work, at which we have arrived is devoted to the—

### MORBID STATES IN WHICH THE CONSTITUENTS OF SECRETIONS AND ALTERED ELEMENTS OF THE BLOOD ARE DISCHARGED WITH THE URINE.

This chapter is, in a great measure, founded on the observations of M. Rayer. Dr. Willis observes, that the number of matters produced by the surfaces of the urinary passages themselves, and by the organs more immediately in relation with these, which are upon occasion discoverable in the urine, is much more considerable than might at first have been supposed. Thus we have scales of the epithelium, mucus, pus, the prostatic fluid, and the spermatic fluid, mingled with the urine. Besides these, we have bile; according to some, milk—and pus, the product it would appear of an inflammatory state of the blood, or of parts or surfaces in a peculiar pathological condition.

A. *Epithelium.*—The mucous surface of the urethra is lined with epithelium, which is constantly throwing off minute plates or squamæ. M. Vigla, has, by means of the microscope, discovered these in multitudes mingled with the urine. The scales, writes Dr. Willis, are easily distinguished; they are exactly like those which may be procured for examination in a moment, by drawing the point of a finger over the inner surface of the lip or cheek, and transferring the moisture to the table-glass of the microscope. They present themselves as small, ragged, generally round, but often irregular, transparent scales, in the middle or towards one side of which a darker and evidently raised point may commonly be distinguished, by which they probably adhered to the mucous surface. These squamæ are sometimes present in such numbers, that they form a considerable part of what is called the mucous cloud of healthy urine.

M. Rayer observes that, in the female, such scales may come from the pudendum; and that he has seen similar ones in urine taken, after death, directly from the calyces and pelvis of the kidney.

B. *Mucus.*—The source of this is obvious. Its quantity varies in the urine of different individuals, and of the same individual at different times. Nay, the quantity of mucus varies in successive portions of the urine voided at one time.

The mucus of the urine, continues our author, when abundant is easily collected for examination upon a filter. When in small quantity, it makes no show upon the surface of the filter, and must be withdrawn from the bottom by means of the pipette. Under the microscope the mucus of the urine presents itself in two forms,—as an amorphous matter, and as a congeries of flattened, rounded, colourless globules, granulated on the surface, and not distinguishable from those of pus, save by the difference of the chemical effect of ether, which digested on pus becomes charged with oily matter, but digested on a quantity of proper mucus-globules remains free from any such impregnation. But we need not add that mucous membranes readily secrete pus, and then the globules gain the coating of oily substance.

M. Rayer makes a variety of observations on the mucus of the urine, which are too elaborate for our pages. The *frothing* seen on agitating the urine is in all probability due to it, and is much increased if albumen also exists in the fluid. As the result of his microscopic observations, M. Rayer assures us, that if we examine as soon as possible after death, the mucus of different portions of the urinary organs, we find:—

1. In the calices and pelvis of the kidney, the mucus often presents small lamellæ or globules, and occasionally both.

2. The mucus of the healthy bladder contains small lamellæ and globules. When its quantity has been augmented by any irritation of the organ, the number of globules is always very great.

3. The mucus of the urethra contains epidermic scales, and very numerous globules during and after gonorrhœa.

4. The mucus is evidently a complex substance, of which a particular principle *mucus* forms the characteristic element, and to which may be added lamellæ, mucous globules, and albumen.

To return to Dr. Willis. He refers for an instant to the tough ropy mucus poured out in great quantities from the inner surface of the bladder, in chronic inflammation of the mucous membrane.

The secretion, he believes, owes several of its distinguishing qualities to this, that it has been chemically acted upon by an alkali, generally the ammonia. In the disease in question, it seems, he remarks, very probable that the exhalents are actually pouring out pus, but that this, by combining at the time of its formation either with ammonia evolved from a portion of urine partially decomposed, or with a neutral salt, such as the chloride of sodium, loses its globular constitution, and beeomes the homogeneous ropy mass we observe.

C. *Pus.*—This is no unfrequent addition to the urine, and its characters should be familiar. Purulent urine, observes Dr. Willis, when voided, is turbid in a degree proportionate to the quantity of matter that is mingled with it. Left at rest the globules of pus speedily subside to the bottom, where they form a well defined stratum of variable thickness. This stratum has generally the ordinary features of pus. When examined under the microscope, pus is found to consist of congeries of whitish globules of twice or more than twice the diameter of the blood-globules, of pretty regular general spheroidal figure, although rugged or granulated on the surface. Purulent urine is always albuminous, but it varies in all other physical and chemical qualities. Sometimes it is of high, in other cases of low specific gravity; sometimes it is highly acid, at other times neutral or positively alkaline; sometimes it is very little disposed to run into putrefaction, at other times it can scarcely be kept for two or three days without undergoing decomposition. When alkalescence and putrefaction have taken place, the pus loses its original and distinctive characters. The globules disappear, and the whole is resolved into a tenacious, glairy, and often semi-transparent mucilaginous looking matter, like thick dirty-coloured white of egg.

But pus is sometimes eliminated by the kidney, independently of any disease of the urinary organs. The recent discovery by Mr. Gulliver, of the existence of globules of pus in the blood, both explains and confirms this fact. Professor Chelius, of Heidelberg observed the urine to become loaded with pus, in a patient labouring under a purulent deposite, within the bag of the pleura, in consequence of a penetrating wound of the chest. The stuff that subsided from the urine could not be distinguished from that which flowed out of the chest by the wound. In puerperal fever, too, a purulent or purulent-looking deposite from the urine is sometimes observed to occur critically. We lately presented a paper by Dr. Mouatt, originally contained in the Calcutta Journal, in which were several well-marked instances of discharge of pus in the urine, in cases of purulent deposits; and we may add, that we have witnessed two such cases ourselves.

D. *Prostatic Fluid.*—This is described by Berzelius, as being clear as water, and susceptible of being drawn out into a thread of a certain length. The fluid which exuded from the cut surface of the prostate of an adult male subject was found by M. Vigla to consist almost entirely of a congeries of very minute globules; and these, or globules presenting the same appearance, he detected on different occasions in the urine; but M. Rayer admits that he knows of no method of distinguishing the prostatic fluid in it.

E. *Spermatic Fluid.*—The spermatozoa, the distinctive peculiarity of semen, have been detected on many occasions in urinary deposites. Dr. Willis remarks:—Once and only once, in one of the cases, were the animalcules found still alive. At all other times they were dead. The presence of these animalculi might possibly give a hint as to the cause of certain anomalous cases now and then encountered in practice, accompanied with great prostration of strength and much emaciation without any recognizable cause. It is possible that the presence of small quantities of the prostatic and spermatic fluids in the urine may have been the cause of the difficulties experienced by chemists in determining the precise nature of certain mucous matters now and then discovered there.

M. Rayer enters much more elaborately into this matter. We do not, however, deem it requisite to follow him, the main facts being,—*that* the spermatic fluid may be mixed with the urine, naturally after coition, an involuntary emission, &c., or, morbidly, under other circumstances—and *that* the detection of the spermatozoa in the urine constitutes, in the majority of instances, the evidence of their presence.

F. *Bile.*—Every body knows that, in jaundice, the urine is tinged with bile. The urine, says Dr. W., is either transparent, or deposites. The sediment may consist of various matters, but all are dyed with bile; sometimes it seems to consist entireiy of yellow flocculi, which remain upon a filter, and prove to be the colouring matter of the bile. A piece of linen dipped in bilious urine and dried, is stained of a bright yellow. Bilious urine having a little hydrochloric acid added to it, commonly becomes of a bright green, sometimes it is turned brown, a variety of effect which appears to depend on the modification under which the colouring matter occurs in the urine. When a quantity of nitric acid is mixed gradually with an equal measure of bilious urine, the mixed fluid becomes first green, then blue, violet, red, and finally yellow, these changes of colour succeeding one another in the course of a few seconds. This phenomenon is distinctive of the presence of the peculiar colouring matter of the bile.

G. *Yeast.*—Dr. Willis concludes the Chapter by stating that, in a case of diabetes, Dr. Prout observed the urine to contain a white milky-like fluid, precisely similar to chyle, which slowly subsided to the bottom of the vessel, and by the rapidity with which the vinous fermentation was induced, seemed to act like yeast. M. Vigla also met with a deposite of this kind, but it was in a vessel that had been used repeatedly to receive the urine of a diabetic patient. M. Quevenne, from a chemical analysis of the matter, pronounced it to be yeast, as energetic as that of beer, and perhaps identical with it. The yeast in these cases was, no doubt, formed by the decomposition of portions of urine which had already sojourned in the vessels from which it was obtained.

We have arrived at the Seventh Chapter of Dr. Willis' work, appropriated to the :—

Morbid States in which Principles foreign to the Urine and the Blood, and derived from none of the Natural Constituents of these Fluids, are eliminated by the Kidney.

This Chapter contains but one section, sweet, though not short,, for its subject is sugar and diabetes. The notions of Dr. Willis upon this subject may be hinted in the following quotation:—

"I allude to the disease generally denominated *diabetes mellitus*, in which the economy appears to be guilty of a kind of felo-de-se, in turning food of all kinds into sugar, which being taken up and circulated with the blood produces effects of a most distressing kind, and finally proves fatal either by wearing out the body, engaged in a hopeless struggle to free itself from a matter which to all intents and purposes acts upon it as a poison, or by inducing incurable disease in some vital organ." 195.

In ordinary digestion, sugar received into the stomach, is converted into alimentary matter; in diabetes, alimentary matter is converted by the system into sugar.

*Melituria* is the term by which Dr. Willis describes *diabetes mellitus*. He mentions a circumstance in reference to the comparative frequency of diabetes which will take some physicians by surprise.

"In this country, and in London especially, it is by no means uncommon; it is often observed in our hospitals; few have practised for any length of time without meeting with an instance or two, and my friend Dr. B. G. Babington has informed me that, on occasion of preparing to keep an *Act* at Cambridge during his college life, his father the late Dr. Babington then in very full practice had found him opportunities of seeing as many as *twenty-three cases of the disease at one time*, in every one of which the sugary state of the urine was ascertained." 197.

Dr. Babington must have been in very full practice indeed to have had twenty-three cases of diabetes mellitus on his own hands at any time. Nor can we credit it. Probably Dr. Babington made interest for his son among his medical friends, and beat up saccharine recruits from all quarters. Even then the muster was large.

We shall merely notice such parts of Dr. Willis's observations upon melituria, as may be striking or useful. It would be idle on our part at this time of day, offering a complete account of the malady. The first point to which we shall allude, is the proven *existence of urea* in large quantity in diabetic urine. Such urine is almost always deficient in lithic acid, and was long thought to lack urea also. But the urea exists, its presence being masked by the sugar, and it cannot be discovered by being made to combine with nitric acid, the ordinary mode of demonstrating its presence, without a peculiar management. Dr. Henry, indeed, by distilling the residue of a given measure of urine at a temperature a little above the heat of boiling water, when urea is decomposed, had ascertained from the quantity of carbonate of ammonia evolved that the characteristic element of urine must be present in considerable proportion at least; but Mr. Kane, by treating the residue of a diabetic urine with dilute nitric acid in a flask, and plunging this into a freezing mixture, obtained fully the average quantity of nitrate of urea. Mr. M'Gregor, again, after getting rid of the sugar by means of fermentation, evaporating slowly, and treating the extract obtained with anhydrous alcohol, &c. found he was able to procure the urea in a crystalline mass and uncombined. In this way Mr. M'Gregor demonstrated that one diabetic patient was passing 1013 grains of urea daily; that a second was voiding 945 grains, a third 810 grains, and a fourth 512.5 grains, the quantity discharged by a person in health amounting to from 362 to 428.5 grains. None of these patients had undergone any treatment, and the specific gravity of the urine in each in succession was 1.040, 1.045, 1.034, 1.050, the quantities of urine discharged being, in the same order, 38½lbs., 30lbs., 40lbs. and 25lbs.

*Relative Quantities of the Ingesta and Egesta in Diabetes.*—It has been a subject of remark, and sometimes of astonishment, that the quantity of urine excreted in diabetes exceeds that of the fluid drunk. But, as Dr. Willis observes, the same circumstance obtains in health. M. Chossat found in the course of his experiments, that during the depth of winter, the quantity of urine excreted by persons in health exceeded their drink in the proportion of 1.5 to 1. Between midwinter and the vernal equinox, the urine fell short of the drink in the ratio of .94 to 1. In the summer months the difference was still greater, the urine falling short of the drink in the ratio of .89 to 1. Even the most solid food consumed by man contains a very large proportion of water; and by whatever amount the urine discharged surpasses the drink imbibed, the excess is in all probability commonly derived from this source. The quantity of dry feces voided by man in the course of the day is extremely small, not amounting to more than from one to two ounces, the individual consuming regularly from twenty to forty ounces of food styled solid in the same time. We also observe sheep, goats, hares, and other herbivorous animals, eating many pounds of succulent food in the course of the day, and discharging only a few ounces, or even a few drams of very hard dry excrement; but they void large quantities of urine, even when they do not drink at all. It is interesting to observe, that the feces in melituria have commonly the dryness and hardness of those of the rodent and ruminating animals mentioned. Dr. Bardsley's inquiries, indeed, may be regarded as militating against these views. With every attention to the weight and measurement of the ingesta, he found that the mass of matter excreted surpassed that taken in. If this were so, the additional fluid was in all likelihood derived from the atmosphere by the mucous membrane of the bronchi; this having been shown in some experiments of Professor Mayer particularly, to have the power of rapidly absorbing fluids placed in contact with it. But, in cases of diabetes, it is difficult to get at the truth in reference to the quantity of the ingesta, patients usually eating and drinking more than the medical attendant is aware of.

*Impotence in a Case of Recovery from Diabetes.*—Dr. Willis alludes to the well-known circumstance, that the sexual appetite generally disappears at a rather early stage of the disorder; and he adds that, in one of the few cases of genuine recovery that he is acquainted with, the only function that remained in abeyance was the sexual.

*The Proximate Cause of Diabetes.*—Dr. Willis sketches successively the opinions or the theories that have, at various times, been formed upon this subject. He is naturally arrested by the experiments and the paper of Mr. M'Gregor, who has established the existence of sugar in the serum of diabetic blood—and shewn its formation in the stomach, the saliva, and the fæces. It is on these observations in particular, that Dr. Willis leans, in announcing the proximate cause of the disease:—

"Derangement of the digestive functions is here unquestionably the head and front of the offence. This derangement too is of a very peculiar kind: the stomach has a disposition to fabricate sugar to a preternatural extent out of the food, particularly the vegetable food, taken into it; for the disease is functional in every part, and is actually *disease* only because the *degree* of a natural process is surpassed—the healthy stomach generates sugar to a limited extent; the stomach of the melituric individual generates sugar in excess. The sugar produced in health is without doubt decomposed in the after-stages of digestion; it does not appear in any of the fluids of the body, nor in any of the secretions save milk, of which it is an element, nor in the feces; in the melituric state the sugar engendered is not decomposed; it enters the circulation. To explain all the rest of the disease,—the fever, and wasting, and excessive secretion of urine, &c., becomes easy after this: there is a substance mingled with the blood which is foreign to its constitution, and which consequently poisons life in its fountain head. The derangement of the kidney is purely secondary, and is consequent upon its efforts as grand purifier of the system along with the lung, to free the circulating fluid of the cause of all the evil—the sugar. The morbid changes discovered in the kidneys after death from melituria, have even generally been extremely trifling, they have mostly been such as indicated simple increase of functional activity; and this, in fact, is all that the kidneys can be rightfully charged with,—if their efforts are not much rather beneficent than prejudicial; for my own persuasion is, that without energetic action on their part, the disease would prove far more rapidly fatal than it does." 218.

The precise morbid state of the digestive apparatus is obliquely glanced at, and furtively dispatched by Dr. Willis. Pathological anatomy has not helped us, the changes that are found being too inconstant and often too inconsiderable to form a stable basis for the erection of a hypothesis.

*Hints for Treatment.*—We shall merely pick out such as are either new, or are more forcibly or ingeniously put than usual. What is pretty well known we shall pass over.

*a.* Dr. Willis remarks:—"*Could we discover any means of preventing the stomach from forming sugar, we should, I believe, succeed in curing the disease.* To this end the efforts of practitioners ought in future, as I imagine, to be directed."

*b.* Dr. Willis reprobates large doses of opium. He conceives that one or two grains of opium in melituria, as in other diseases where the medicine is decidedly indicated, are in general the proper dose; if we can do with half a grain, so much the better. It is very commonly advantageous to aid the natural tendency of opium to the skin by giving it a combination with ipecacuanha, or still better with minute doses of the tartrate of antimony. This latter, he fancies, allays the craving at the stomach.

*c.* He naturally lauds the sudatorium. It happens, he says, very rarely indeed that a melituric patient is found who resists this powerful agent, which causes large drops of sweat to start upon the skin where no trace of moisture had been seen for months. The sudatorium has even been held as a complete prophylactic of the disease we are discussing; and it is curious to learn that in Russia, as Dr. Lefevre informs us, where every peasant and artizan makes use of the sudatorium, the disease is unknown. He had himself looked in vain through the records of the principal civil and military hospitals of the Russian empire, for the mention of a single case; and Sir James Wylie had not met with it once among upwards of two millions of soldiers who had passed under his notice as general military inspector. But the sudatorium, though seemingly useful, ultimately failed in two cases of Dr. Watson's. Still it is a valuable adjunct to our remedies.

*d.* He seems to anticipate much from magnesia. It has undoubtedly, he observes, been several times employed with more of what, in opposition to the prevailing views of the day, might be termed a *specific* effect, than any other medicine. It is one of the very few articles too that has done good in the hands of different practitioners. Hufeland, among others, speaks highly of its powers. Mr. Benjamin Phillips lately prescribed magnesia upon two occasions to the same individual labouring under melituria in an early stage, and in each instance with success: the sugar disappeared from the urine, the thirst and all the other symptoms of the disease were immediately relieved and speedily vanished.

*Plan of making nourishment less nutritious.*—Dr. Willis, observing that Nature has mixed the food of all but the pure carnivora, with a large proportion of insoluble and innutritious matter, would act on this hint in a case of diabetes. He would supply a moderate quantity of nutritious animal food,—yolk and white of egg, beef and mutton finely chopped, soft curd, &c., mixed with a considerable proportion of woody fibre and indigestible matter, such as bran freed from all amylaceous particles, by boiling in a large quantity of water, the woody part of the potato separated from its starch by rasping and washing, and the like.

Dr. B. G. Babington has observed that green vegetables—the oleracea, such as spinach, cabbage, celery, &c., may be taken without increasing the saccharine qualities of the urine. These vegetables include next to no amylaceous particles; they, however, include a considerable proportion of gummy extractive matter, upon which their nutritive qualities entirely depend. If it be found, says Dr. W. that these are not converted into sugar in the stomach, the discovery would be important. We have our doubts.

*Proposal to exclude Insalivation.*—Dr. Willis, in his laudable anxiety to seize on any remedy for so desperate a disorder, as drowning men are known to catch at straws, proposes to exclude, so far as may be, the action of saliva from the process of digestion. He does this, because the experiments of Leuchs, Schwann, and Mueller have shewn the power possessed by saliva of converting starch into sugar.

"Would any thing be gained by administering food to patients affected with melituria, without suffering it to undergo the ordinary preliminary mastication, thereby preventing it from being mingled with saliva? Would food that has not been mixed with saliva, thrive with man for any length of time? I believe it would." 241.

"Could vegetable food be administered to melituric patients without prejudice through an œsophagus tube? If the agent in the conversion of fecula into sugar be saliva, as seems certain from the experiments of the distinguished physiologists referred to, we should expect to avoid this conversion by preventing any saliva from reaching the stomach. Chemistry, which has served medicine so efficiently and on so many occasions, ought surely to come to her aid in this instance, and show as well what will *prevent* as what will *cause* the formation of sugar from starch." 242.

We fear, in the first place, that the attempt to exclude saliva from the stomach during digestion would prove in the end abortive; and, in the second place, that the formation of sugar hinges on something deeper than any such remedy, were it practicable, would affect. Still, the proposition having an experimental basis, may perhaps, deserve a trial.

*Are Diabetic Patients Intellectual?*—It has been remarked, says Dr. Willis, that those who suffer from diabetes are generally individuals of superior intelligence. Hufeland, writing after he had passed something like sixty years in the exercise of his profession, says he never met with a *stupid* man affected with diabetes. From what we have seen of diabetes, it has appeared to us that individuals affected with it have been much like the ordinary run of mortals, some clever and some obtuse. Of this we are certain, that one of the *stupidest* of clod-hoppers died of it under our observation. Hufeland's, we should conceive to be one of those whimsical Continental crochets, that make plain folks on this side of the water stare.

The eighth Chapter of Dr. Willis's interesting work is on the

### FORMATION AND GROWTH OF URINARY CALCULI.

As most of what Dr. Willis says *must* be familiar, we shall continue to adopt the plan of selecting only what is particularly striking, or novel.

*Mode of giving Alkalies for Lithic Acid Gravel.*—Dr. Willis, after considering the caustic alkalies as nauseous, if not acrimonious (a censure which is perhaps too harsh and indiscriminating), and approving, of course, of the bicarbonates of potash and soda, observes that it is of great importance to exhibit them in a state of very plentiful dilution. Equal quantities, he says, of some of the natural mineral waters that actually contain but one or less than one part of bicarbonate of soda in 200 of the fluid, are more potent in rendering the urine alkaline than a solution of the same salt in the ratio of one to fifty or sixty. A dram, or at most two drams of the salt in from a pint and a half to three pints of fluid, is the proper proportion; and this quantity may be taken at intervals in the course of the day, with the effect of powerfully increasing the activity of the kidneys, speedily putting an end to the acidulous state of the urine, and either washing out lithic grit and concretions of late formation from the pelvis of the kidney, from the ureter or bladder, or disintegrating and dissolving them, wherever they chance to be arrested. Should the urine not become neutral in the course of a day, the quantity of the alkali may be increased. That the soda may be thus taken for a great length of time with impunity, several remarkable cases prove, though we think that this should not lead to the indiscriminate exhibition and abuse of a valuable medicine.

"The only improvement," he continues, "I can suggest on the mode of taking the bicarbonate of soda above recommended, were, in case circumstances could be made to bend to such a plan, to advise the sufferer from gravel to take it at the well-head in one or other of the natural mineral waters in which it is contained. The mineral waters of Vichy in particular, which hold in solution about one part of bicarbonate of soda in 200 of the menstruum, quickly render the urine of the drinker neutral or alkaline, and afford great relief to the unhappy sufferer with gravel and urinary calculus. The waters of Selters, Obersalzbrunn,* Aix la Chapelle, Pouges, Spa, Carlsbad, &c., also contain carbonate of soda in variable and smaller quantities, and have long obtained and deserved much celebrity for their virtues in certain forms of gravelly complaints. One of the pleasantest sparkling waters I met with in a foot ramble through Germany, now many years ago, was in the immediate vicinity of Carlsbad." 259.

Dr. Willis supposes that under diluents with the alkaline carbonates, a lithic acid calculus descending from the kidney to the bladder, may be actually disintegrated and dissolved. This certainly may be the case, but it is evidently difficult of proof. Dr. W. relates two cases where the symptoms of a calculus descending the ureter were not followed by the expulsion of any. But as the patients did not die, the evidence is of course defective, and although such cases may render the fact probable, they do not, and they cannot demonstrate it.

Dr. Willis dwells very forcibly and very properly, on the necessity of not being put off our guard by the lull of symptoms which follows the entrance of the stone into the bladder. The calm, he says most truly, will certainly impose upon the patient, uninformed as he must almost necessarily be in regard to the nature, progress, and tendency of his disease; but it ought not to lull his medical adviser into a security that is most dangerously indulged in for a day. The fact is, that this is just the time when the medical man *should* be on the alert. He has probably been consulted for the sharp symptoms of the passage of the calculus from the kidney—he will soon cease to be consulted, by reason of the temporary calm—when he is again consulted, the calculus will probably have attained a size that makes the case of a widely different complexion.

*Alternating Deposition of Lithic Acid and Phosphatic Calculi.*—Dr. Willis observes:—

"It has generally been admitted as a law, on the high authority of Dr. Prout, that the phosphatic diathesis once established, that is the triple phosphate of ammonia and magnesia and the phosphate of lime, being once deposited on a calculus, matter of another kind is never deposited upon it afterwards. I imagine that this law is not quite so general. If ordinary physical characters may be trusted at all, I have seen calculi in which laminæ of the phosphates were en-

* "See an interesting case of nephritic calculus brought away, and a long period of suffering from gravelly complaints completely removed by the use of this water, in the person of Dr. Lebenheim, of Trebnitz, related by himself, in vol. xlix. of Rust's Magazin. He voided as many as twenty calculi of different sizes at once. The pelvis of one if not of both kidneys must have been full of concretions."

veloped by others of lithic acid or the lithates, and even of the oxalate of lime. These instances, however, were in public or private collections, and so circumstanced that I had no opportunity of putting the matter to the test of experiment by an analysis. Among authors on the subject, indeed, I find many examples in entire contradiction to such a law." 293.

And he quotes one case from Marcet, and three from Brugnatelli, which, so far, bear him out.

Here, we believe, we must pause. In our next number, we shall resume, and conclude, for a season, the subject of Diseases of the Urinary Organs. To M. Rayer we shall turn for a history of the acute and chronic inflammation of the kidney, complete, and excellent, and instructive in the very highest degree. It is hardly necessary upon our parts to press our readers very urgently to study these complaints.

